# Supplementary material for: Mice in social conflict show rule-observance behavior enhancing long-term benefit
Source: Nat Commun. 2017 Nov 7;8:1176. doi: 10.1038/s41467-017-01091-5 (PMC5673895; doi:10.1038/s41467-017-01091-5)
Supplement: Supplementary file 1 — Supplementary Information [file 41467_2017_1091_MOESM1_ESM.pdf]

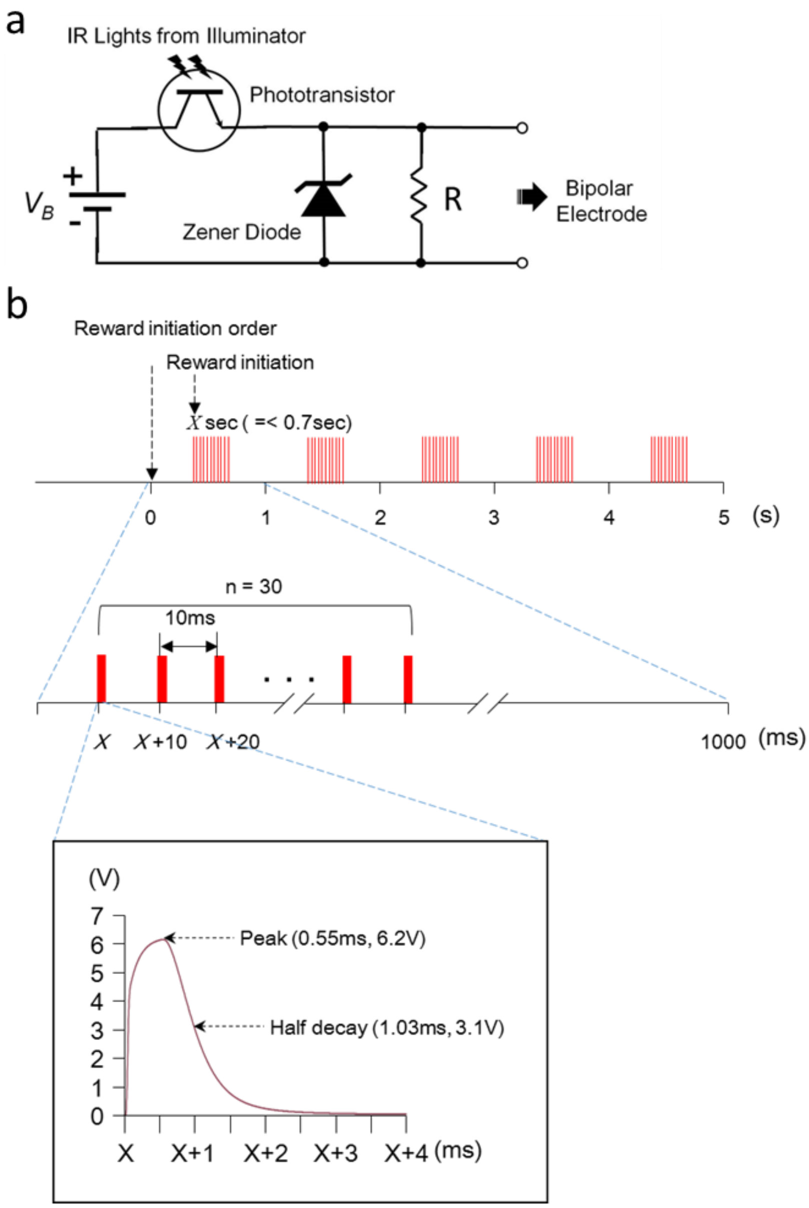

Supplementary Figure 1. Wireless electrical brain stimulation (WBS). a, Scheme of WBS-headset. The headset equipped a battery pack (V_B; 12V), a photo-switch (phototransistor), a Zener diode (breakdown voltage is 6.2V and a 47KΩ resistor (R). Infrared light from the illuminator opens the photo-switch. Following, maximum 6.2V is charged between the ends of bipolar electrodes. **b,** Trains of electrical pulses. The first train was initiated within 0.7s after the initiation order. We used five trains of electrical pulses as one WBS-reward (1 train per 1 second). Each train comprised with 30 individual pulses. When we sent an infrared light for 0.2ms, an electrical pulse was charged at the bipolar electrodes. Test impedance was 70KΩ. The pulse reached to the maximum amplitude (6.2V) at 0.55ms after sensing the IR light and dropped to the half of maximum (3.1V) at 1. 03ms.

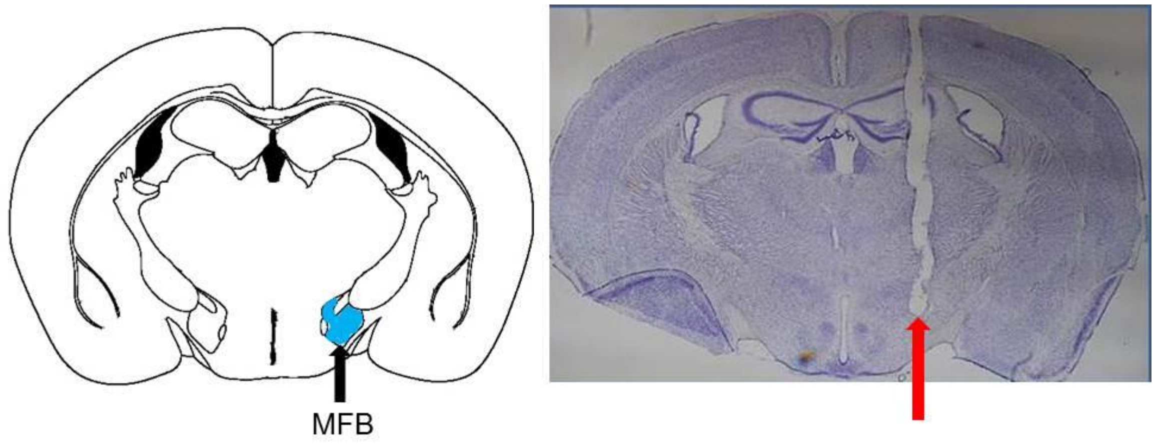

Supplementary Figure 2. The location of the medial forebrain bundle (MFB). We targeted the MFB in electrode implantation. The red arrow indicated an actual insertion point of electrode.

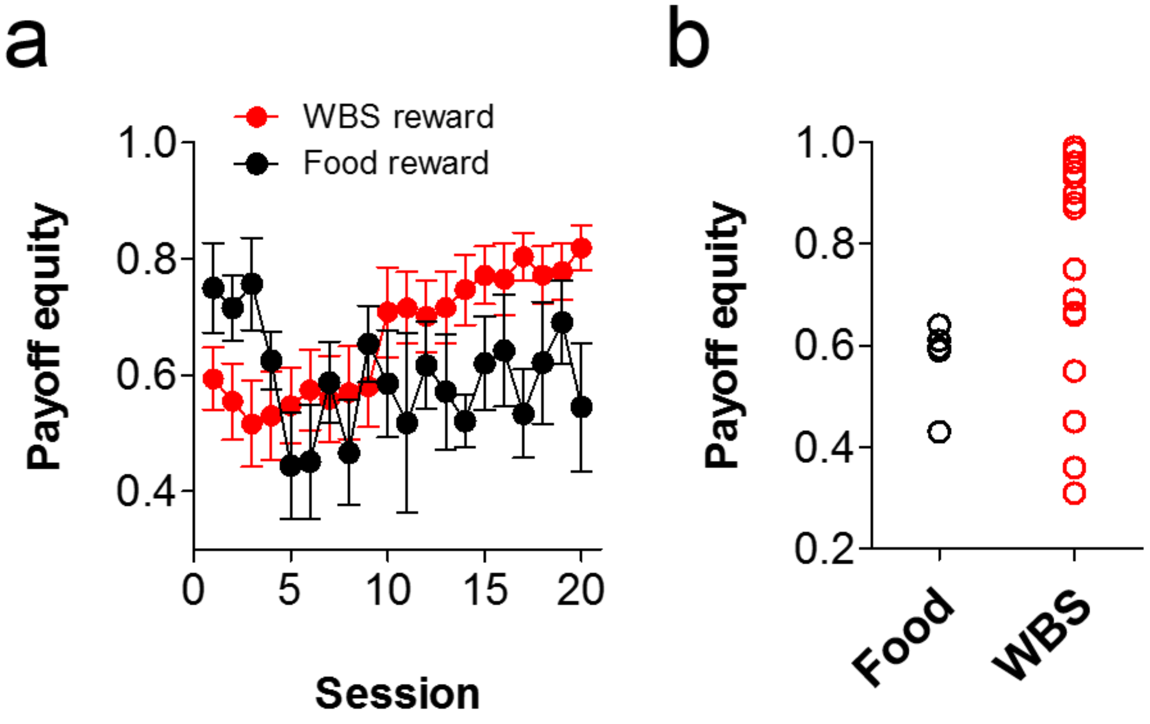

Supplementary Figure 3. Payoff equity increased in WBS condition. a, Payoff equity, the payoff acquisition ratio of one mouse (who obtained less) over the other mouse (who obtained more), increased in the WBS condition (Red circle, Friedman RM ANOVA on Ranks, x^2 = 61.1, d.f. = 19, p < 0.001), but not, in the food condition (Black circle). **b,** The variance of payoff equity was significantly different between the two conditions (* p < 0.05, f-test).

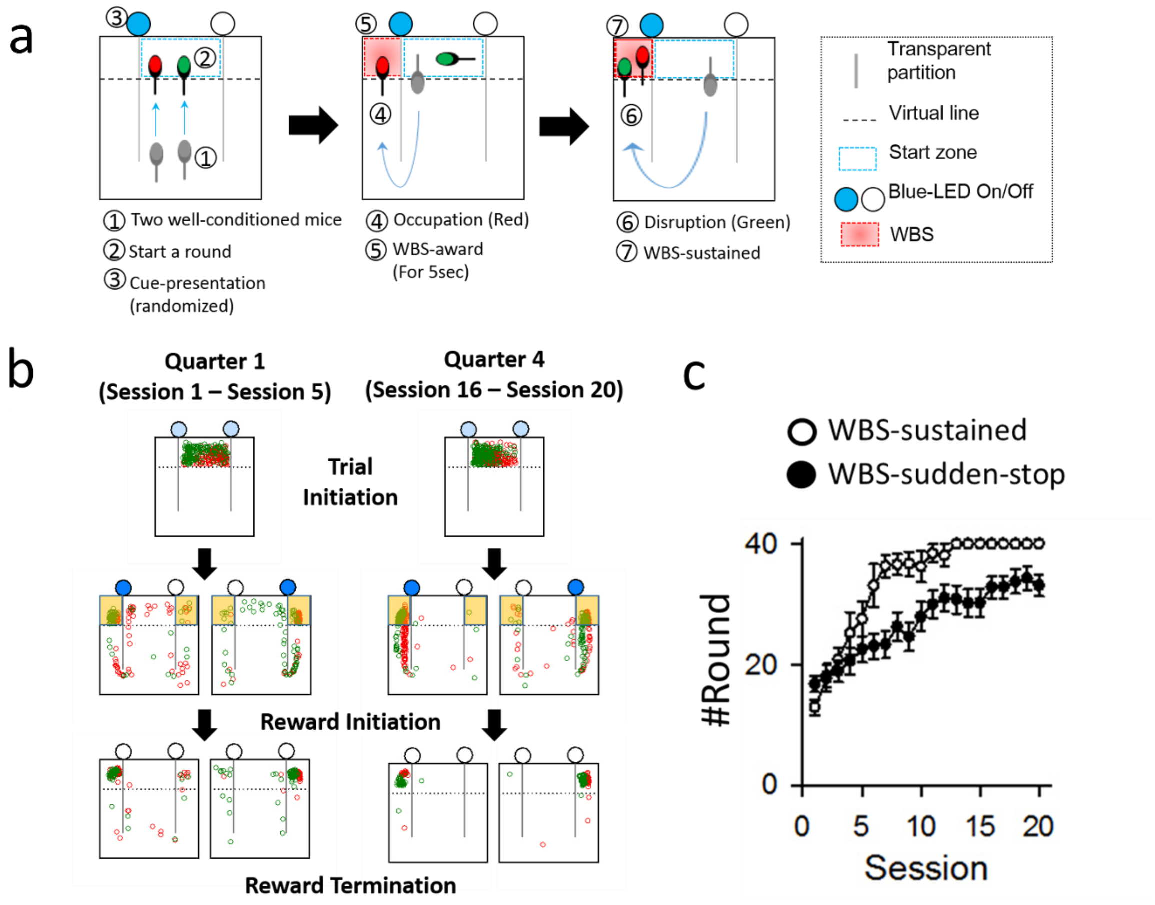

Supplementary Figure 4. Control experiment to confirm that mice perceive that the amount of WBS reward is decreased due to the disruption by the opponent. a, A behavior task protocol that allows full duration of WBS reward regardless of disruption. **b,** Pictorial presentation of the position of the mouse at given time points. Each circle (Green, M_R; red, M_L) indicates the position of a mouse at the designated time point, trial initiation, reward initiation, and reward termination. Left panel shows summation of the first quarter, from session 1 to 5. Right panel, the last quarter, from session 16 to 20. **c,** The number of rounds played per session with the modified protocol increased significantly faster than that with the original protocol.

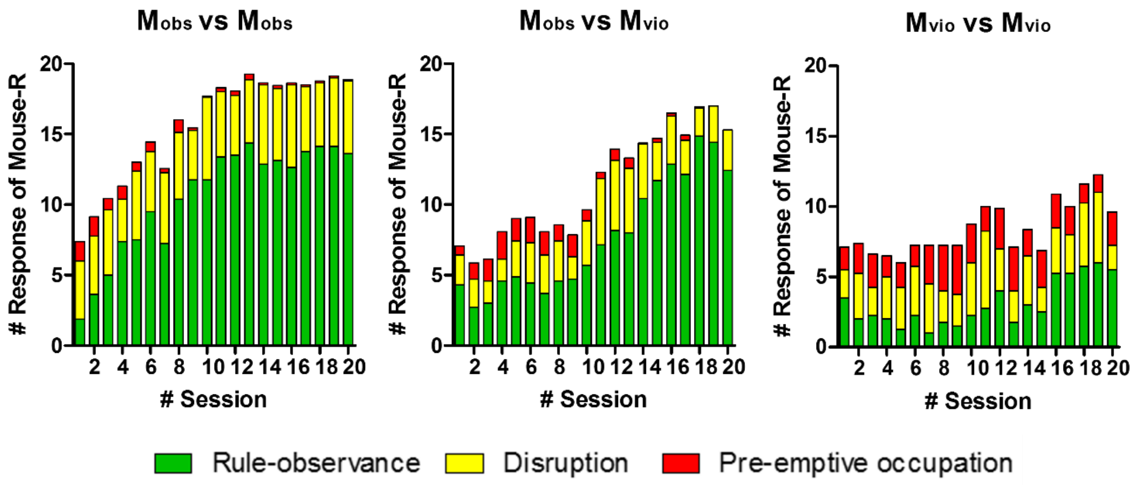

Supplementary Figure 5. Evolution of the response type of the M_R through the sessions for each of the three pair types. Green bar, rule-observance; Yellow bar, rule-violation (disruption); Red bar, rule-violation (pre-emptive occupation).

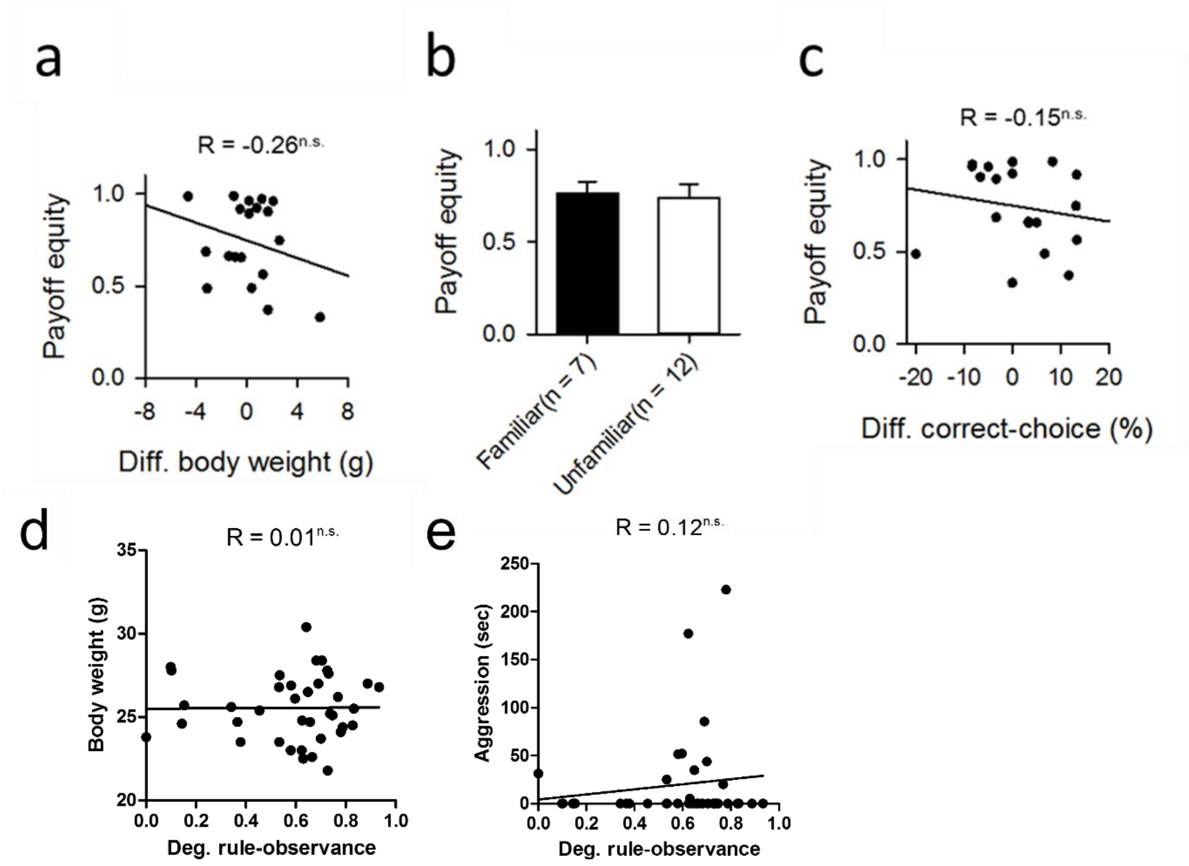

Supplementary Figure 6. Payoff equity and disparities in individuals. a, The difference in the body weight between the mouse who obtained less payoff and the other mouse who obtained more payoff (Diff. body weight) was not associated with payoff equity. **b,** Payoff equity between the familiar pairs and the unfamiliar pairs was not different. **c,** The difference in the correct-choice rate between the mouse who obtained less payoff and the other mouse who obtained more payoff (Diff. correct-choice (%)) was not associated with payoff equity. **d,** The body weight shows no correlation to the degree of rule-observance. **e,** Duration of aggressive interaction was not associated with the degree of rule-observance.

Supplementary Figure 7. Pictorial presentation of the position of the mouse at the reward initiation time for each sessions, for each of the 19 pairs.

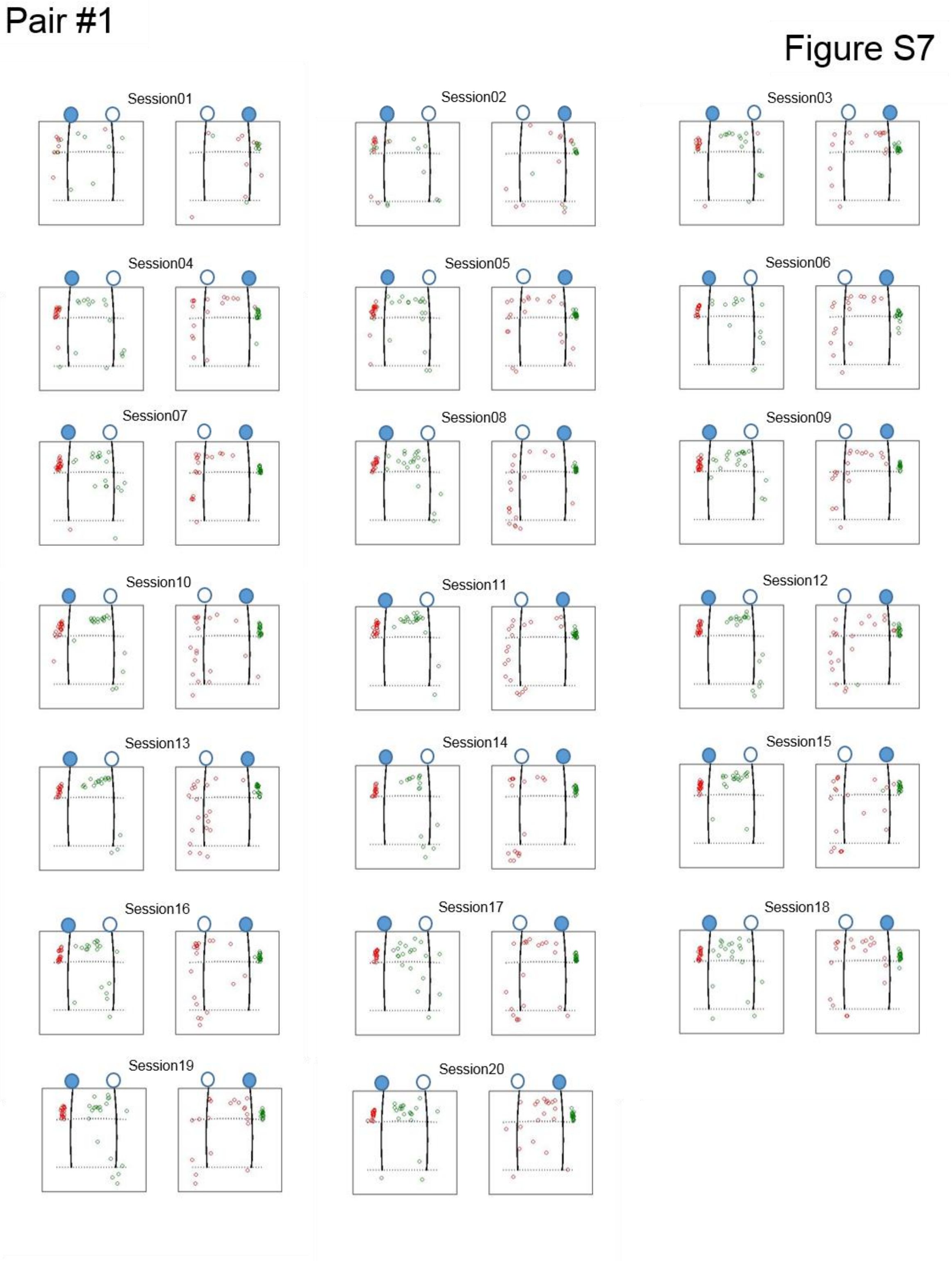

Pair #2

Figure S7

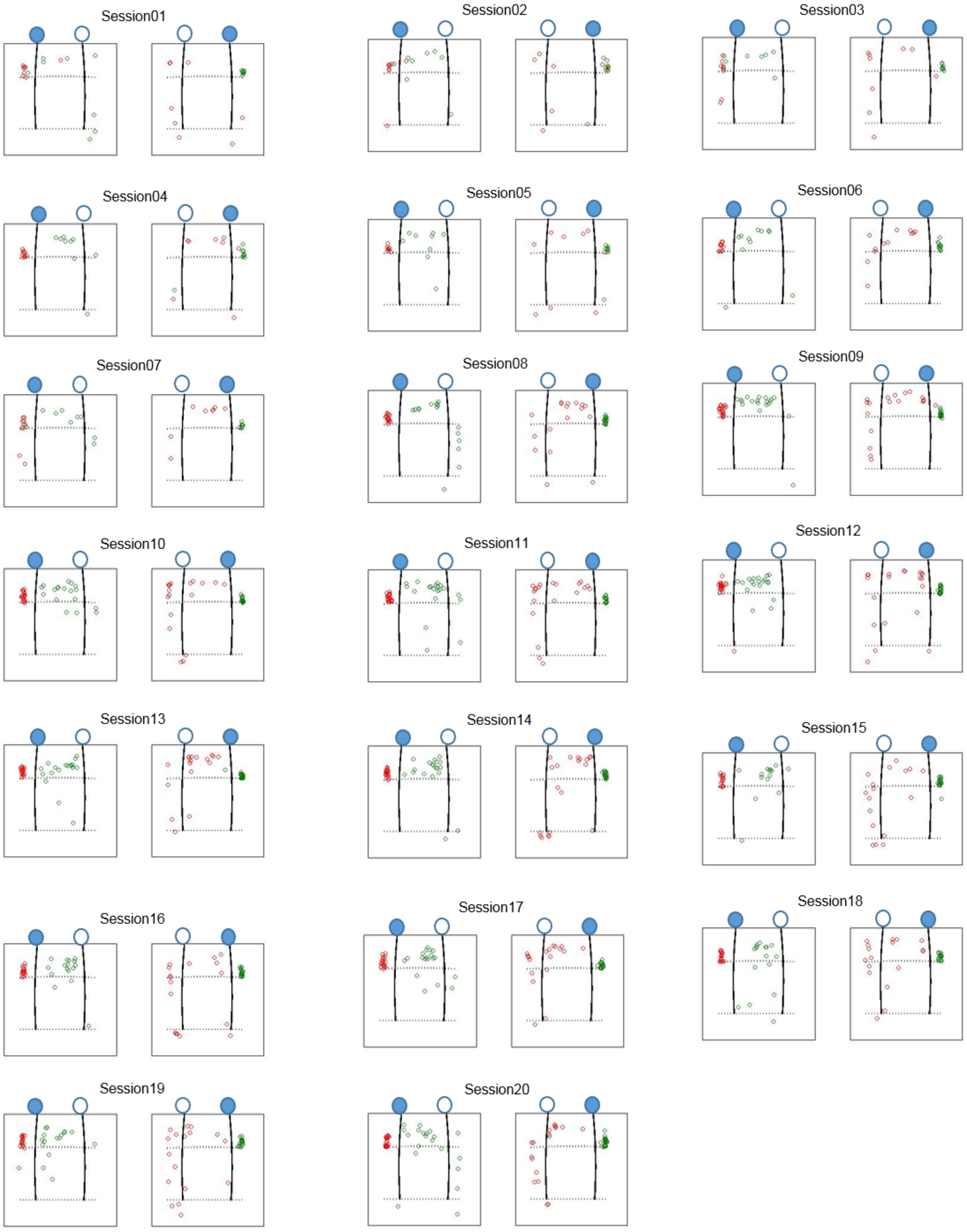

Pair #3

Figure S7

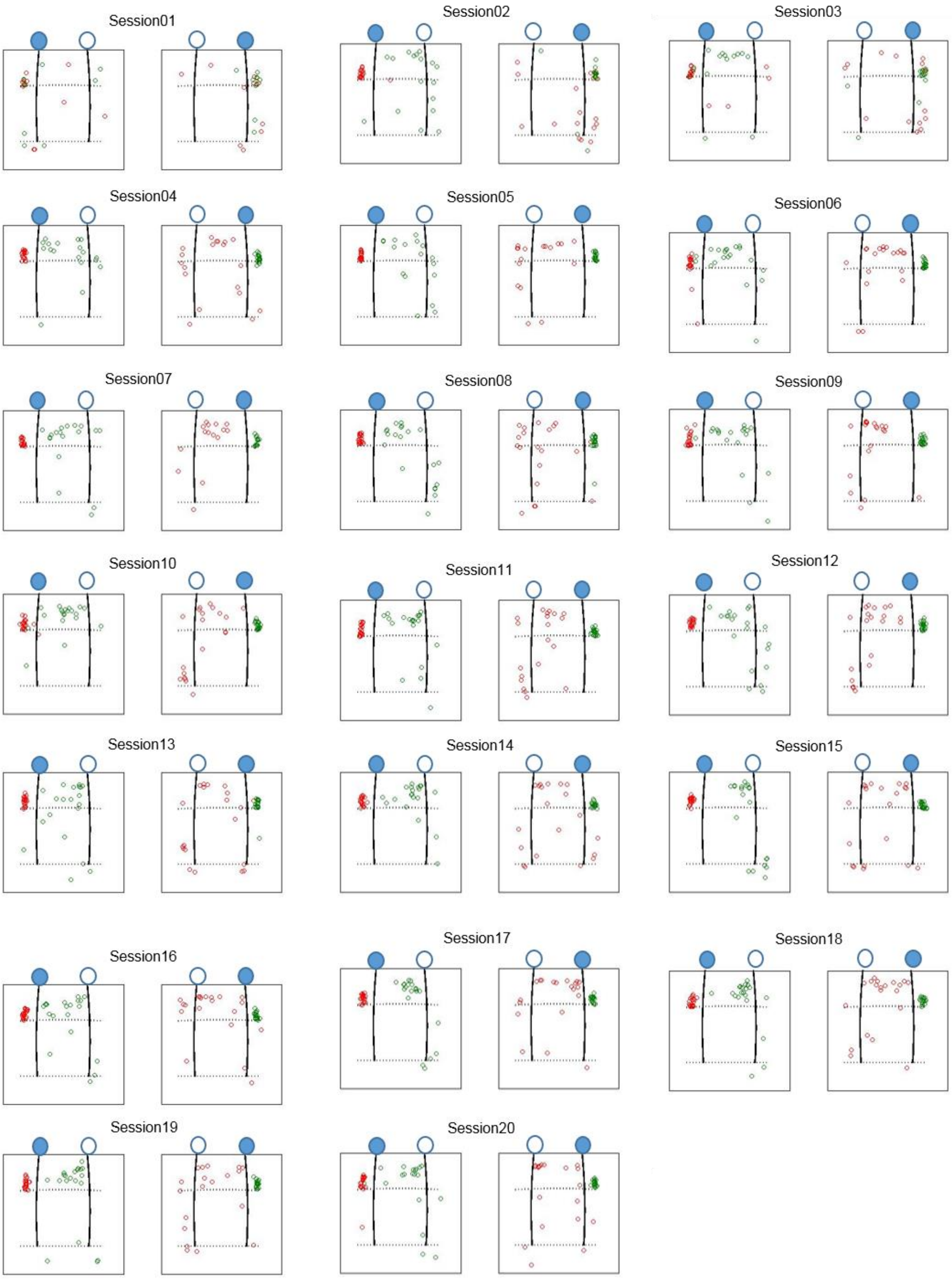

Pair #4

Figure S7

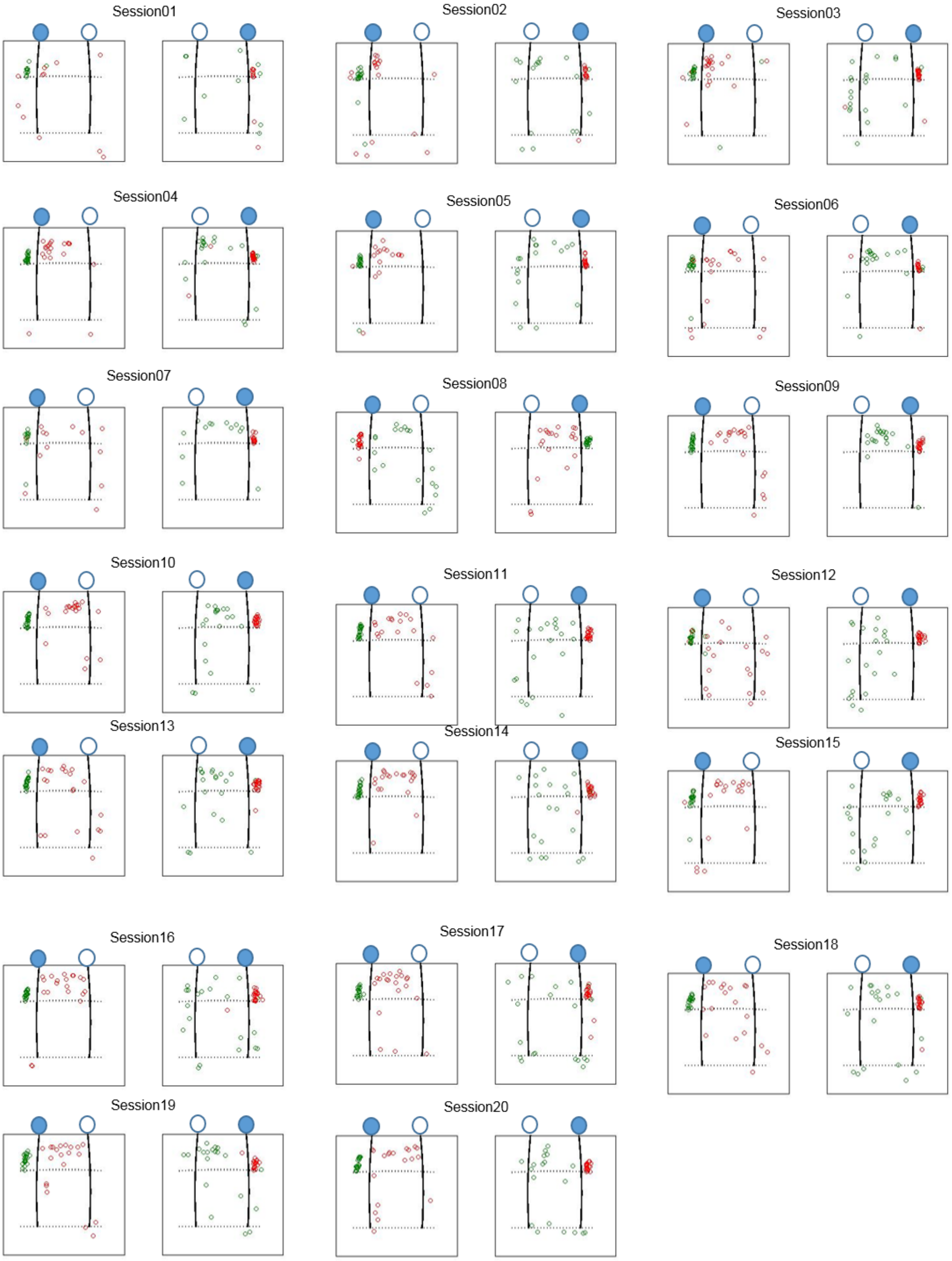

Pair #5

Figure S7

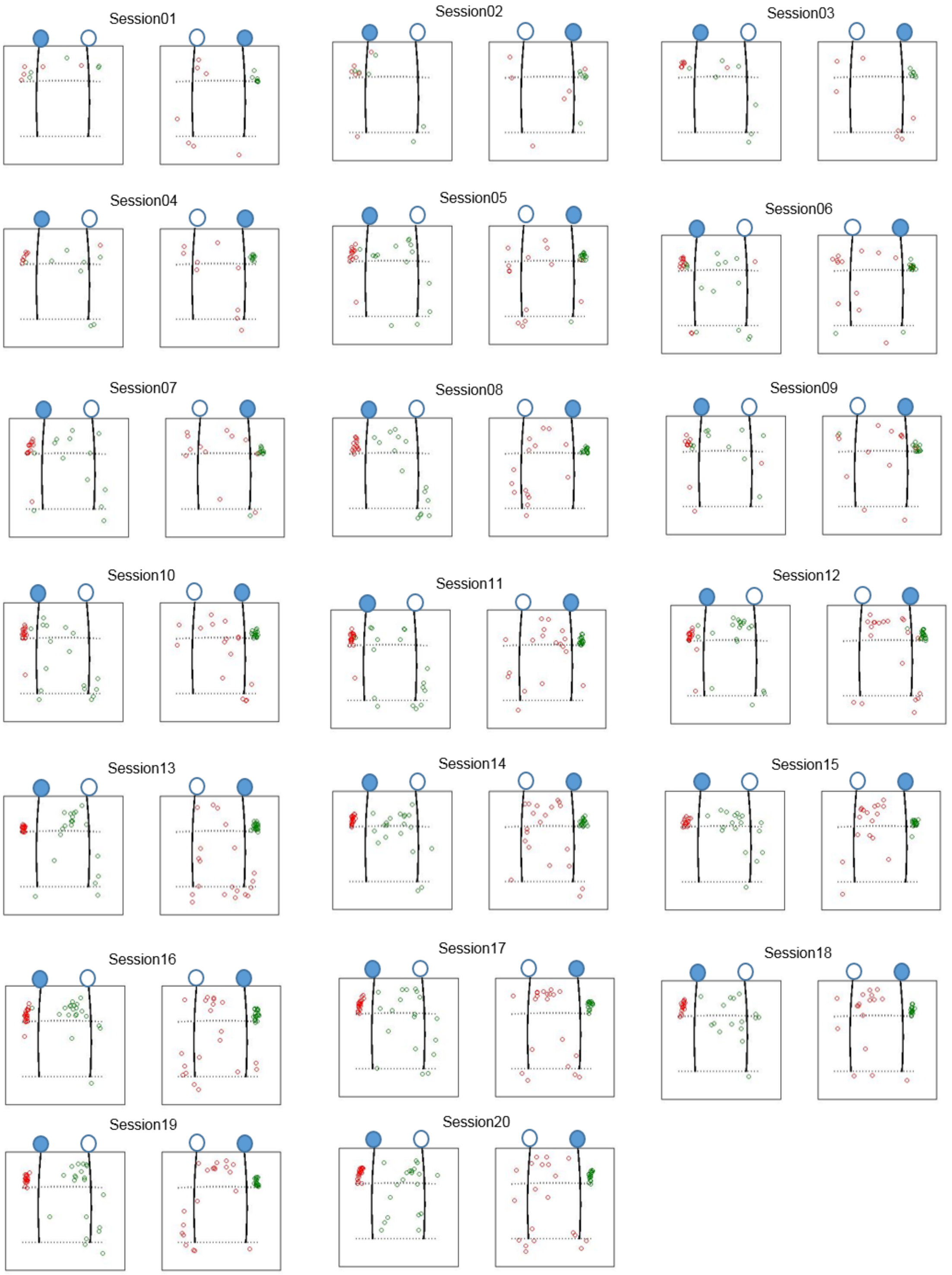

Pair #6

Figure S7

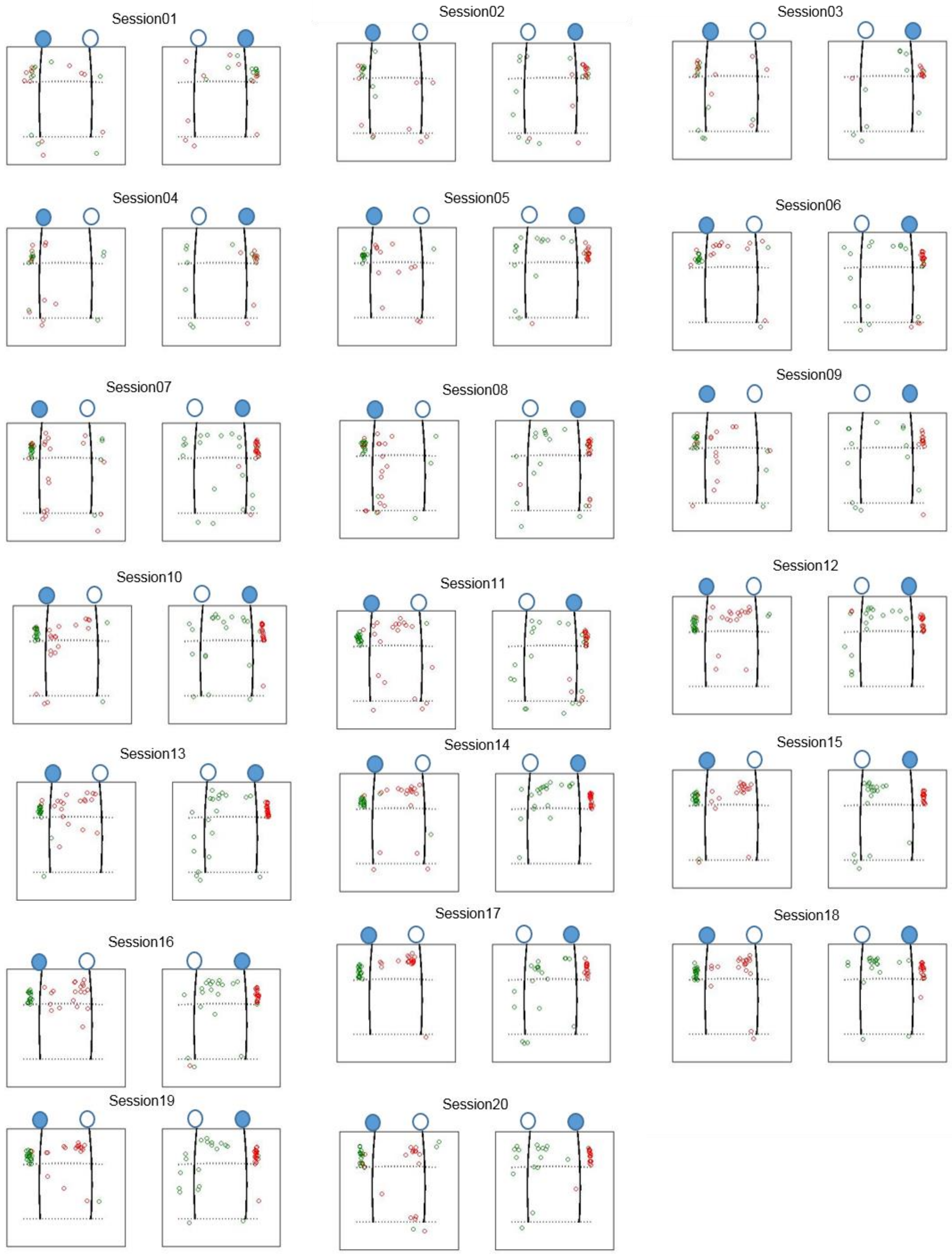

Pair #7

Figure S7

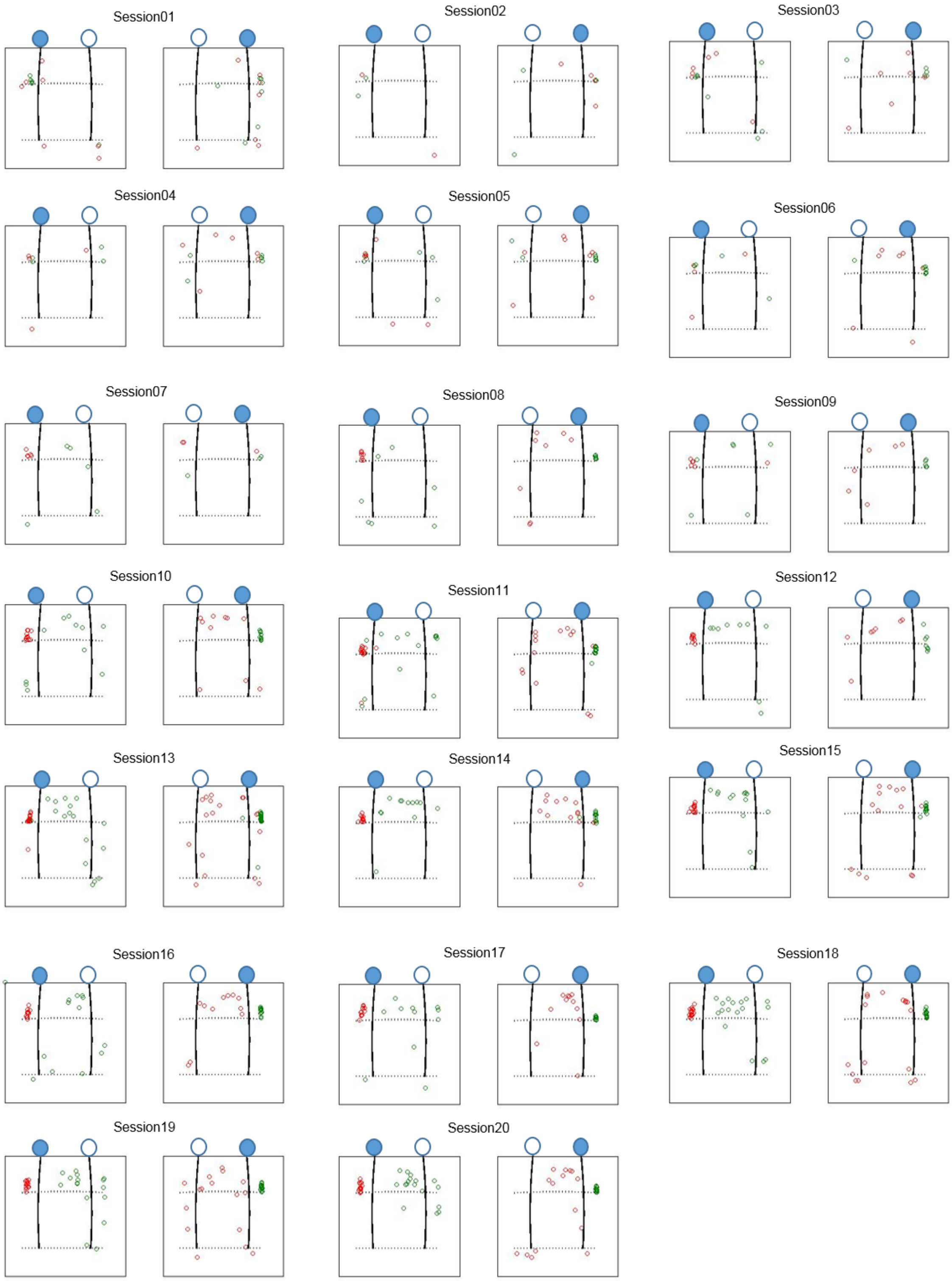

Pair #8

Figure S7

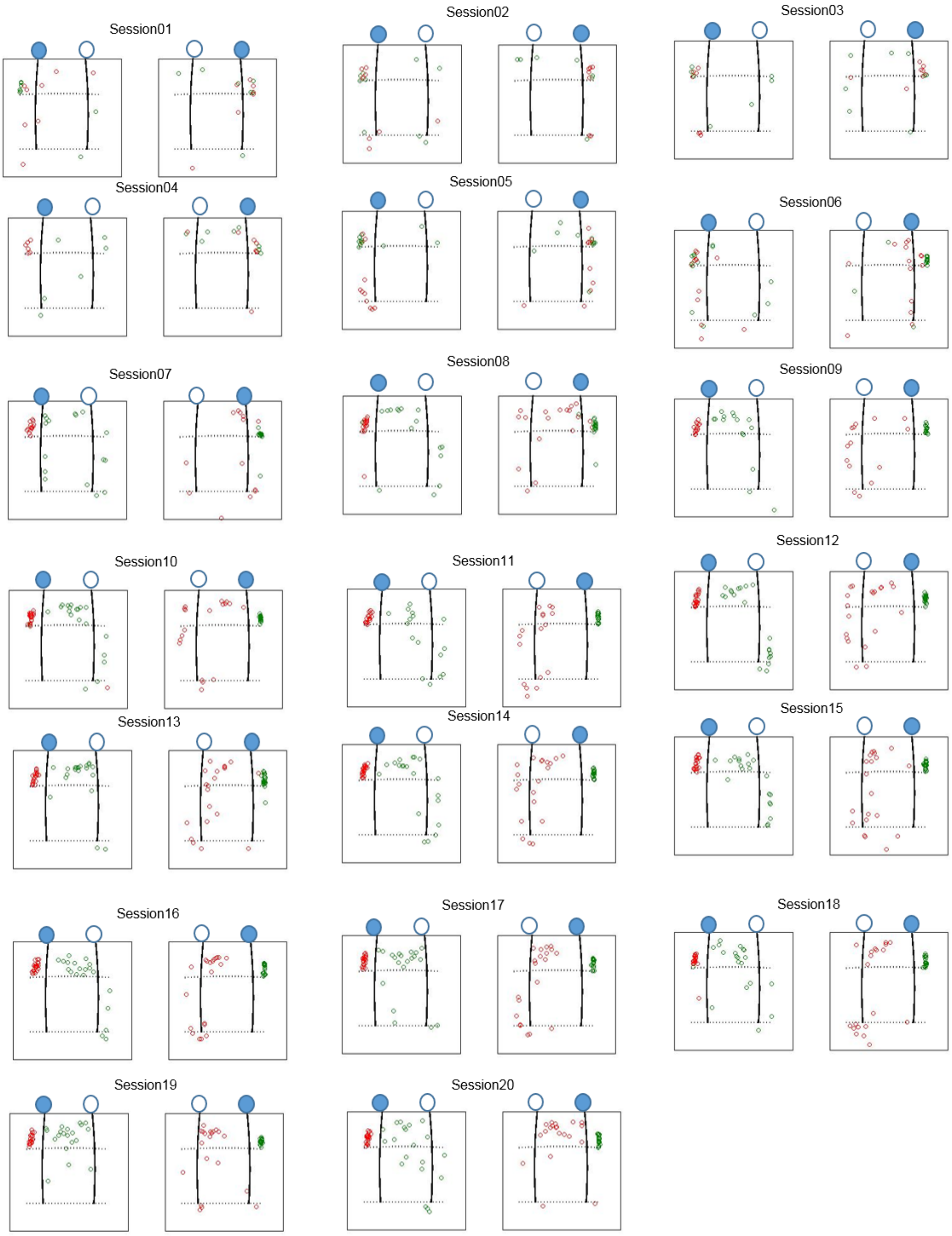

Pair #9

Figure S7

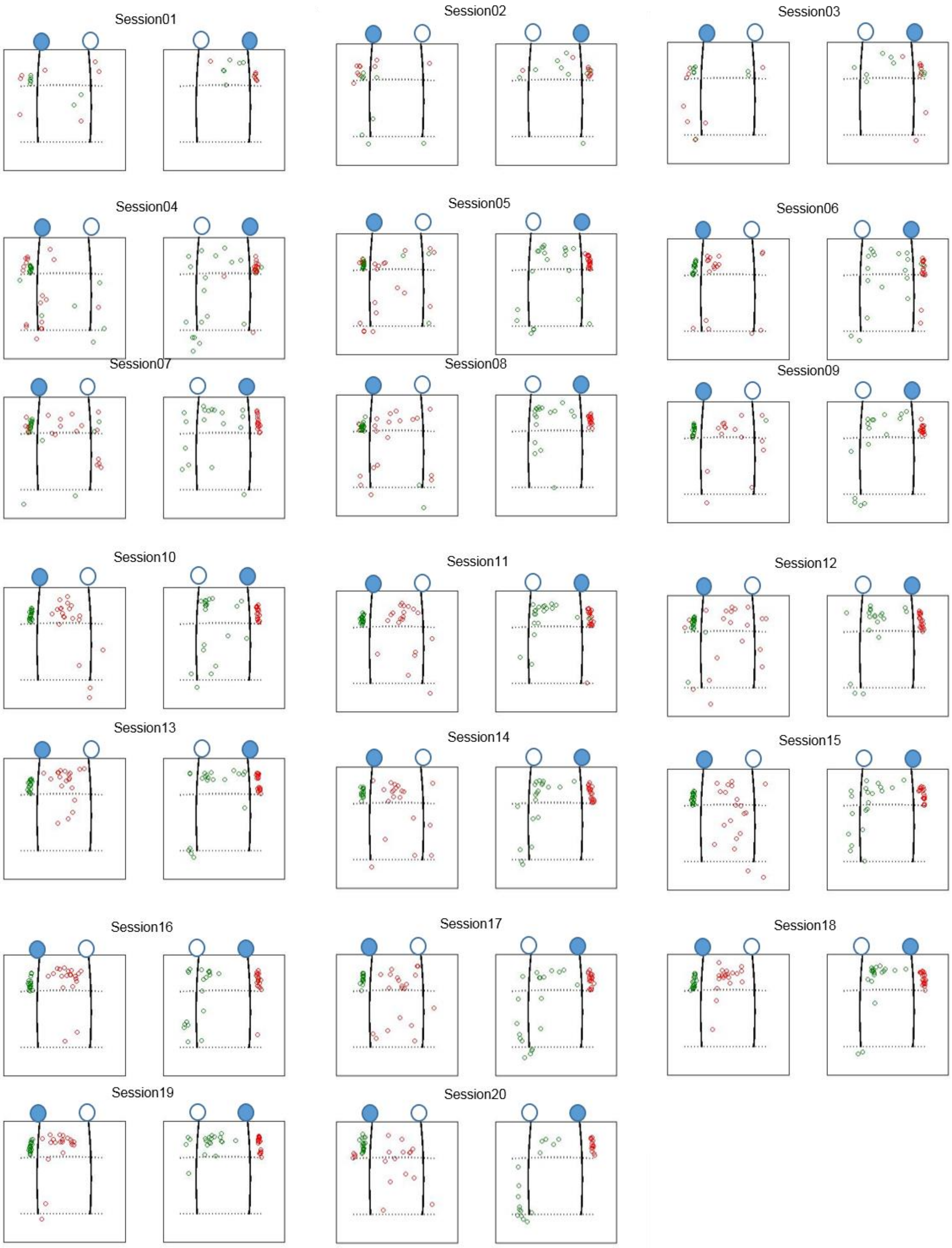

Pair #10

Figure S7

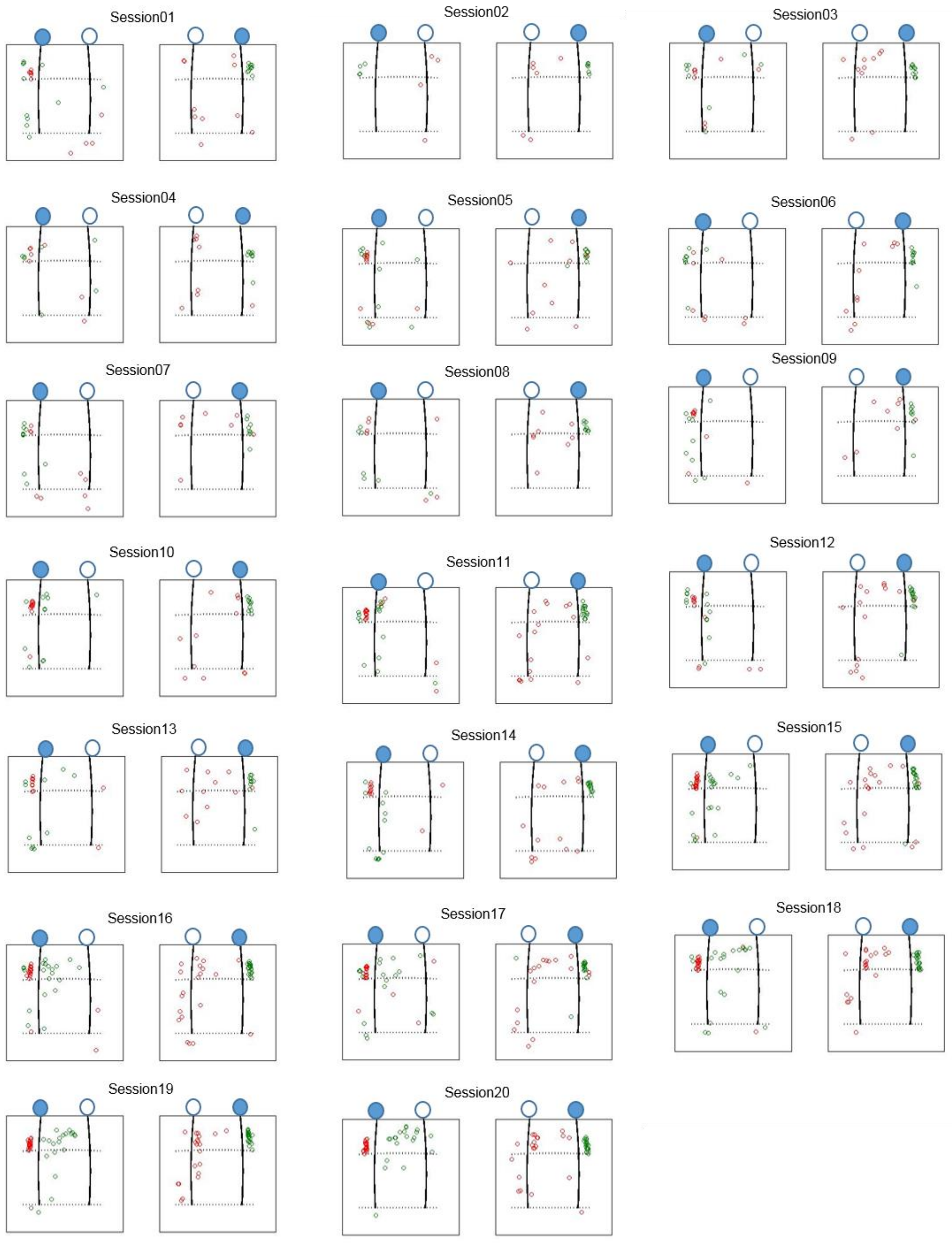

Pair #11

Figure S7

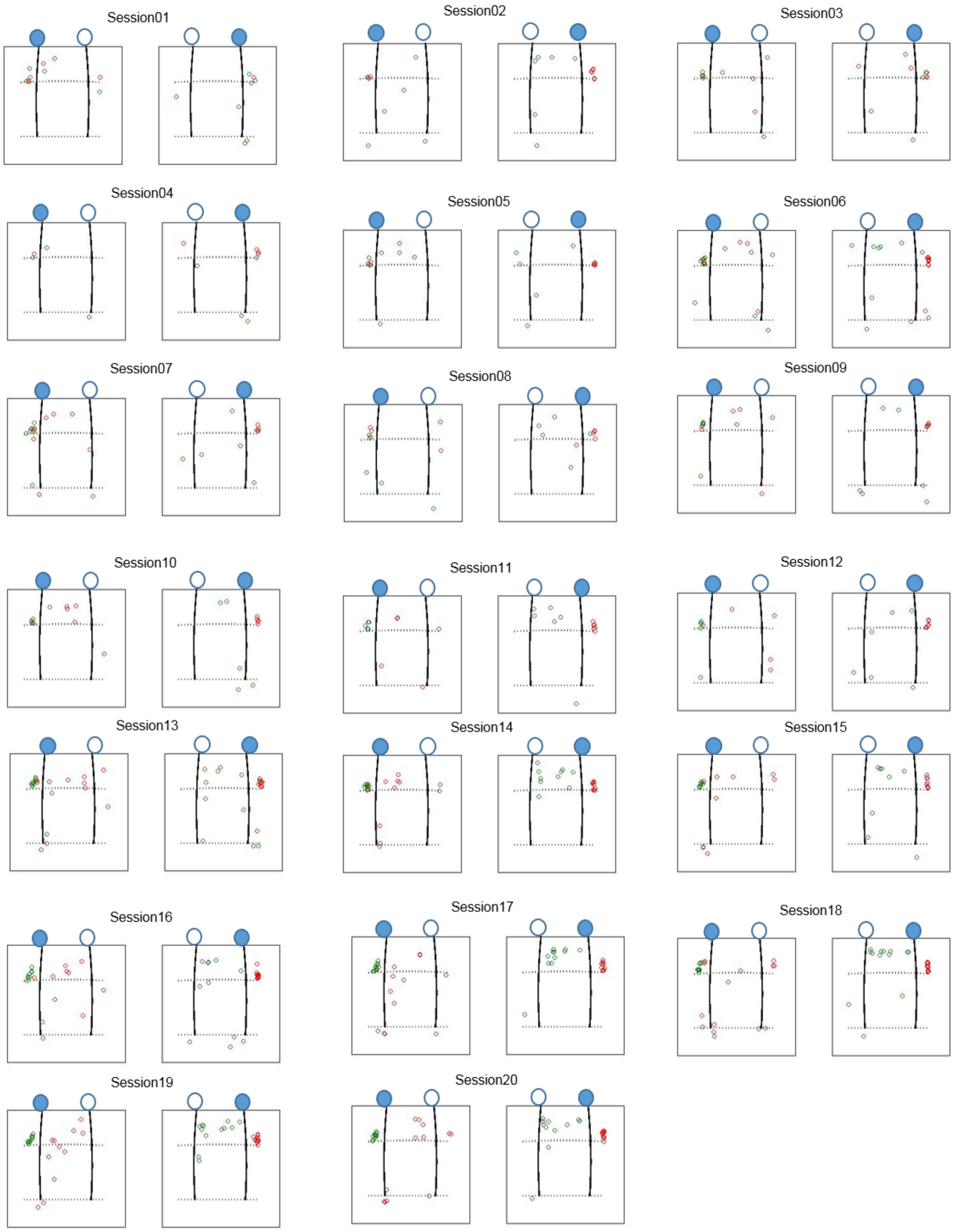

Pair #12

Figure S7

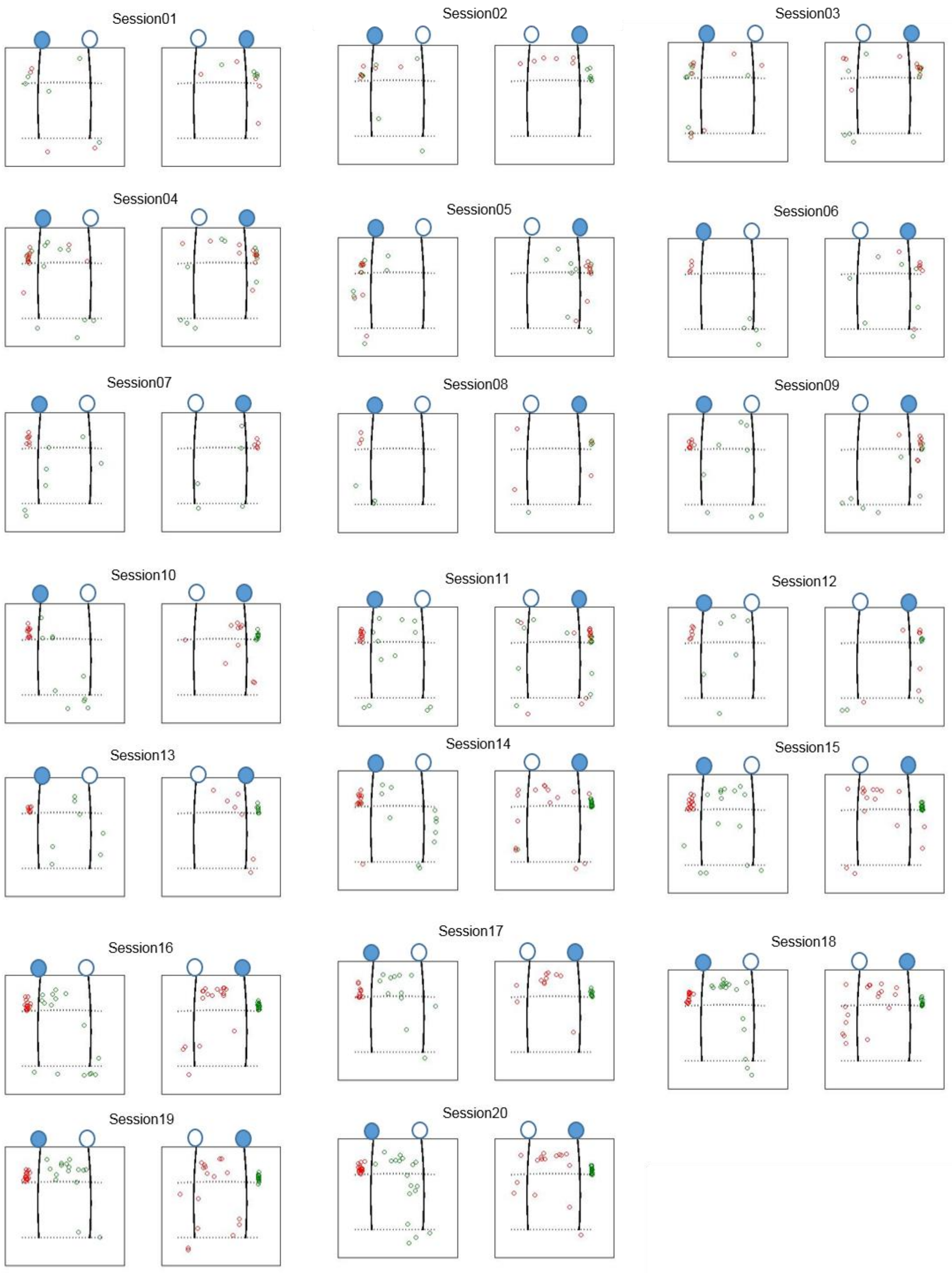

Pair #13

Figure S7

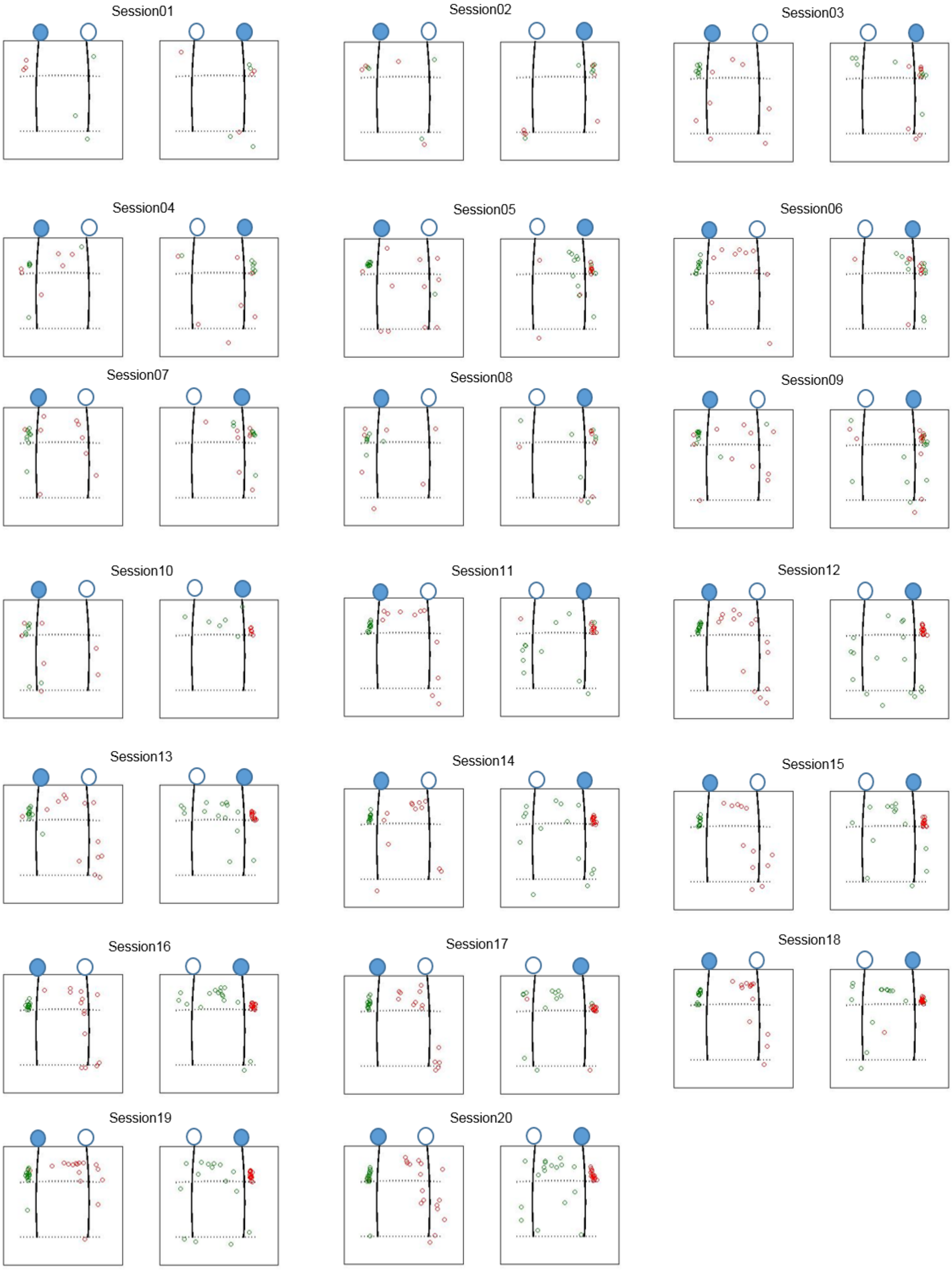

Pair #14

Figure S7

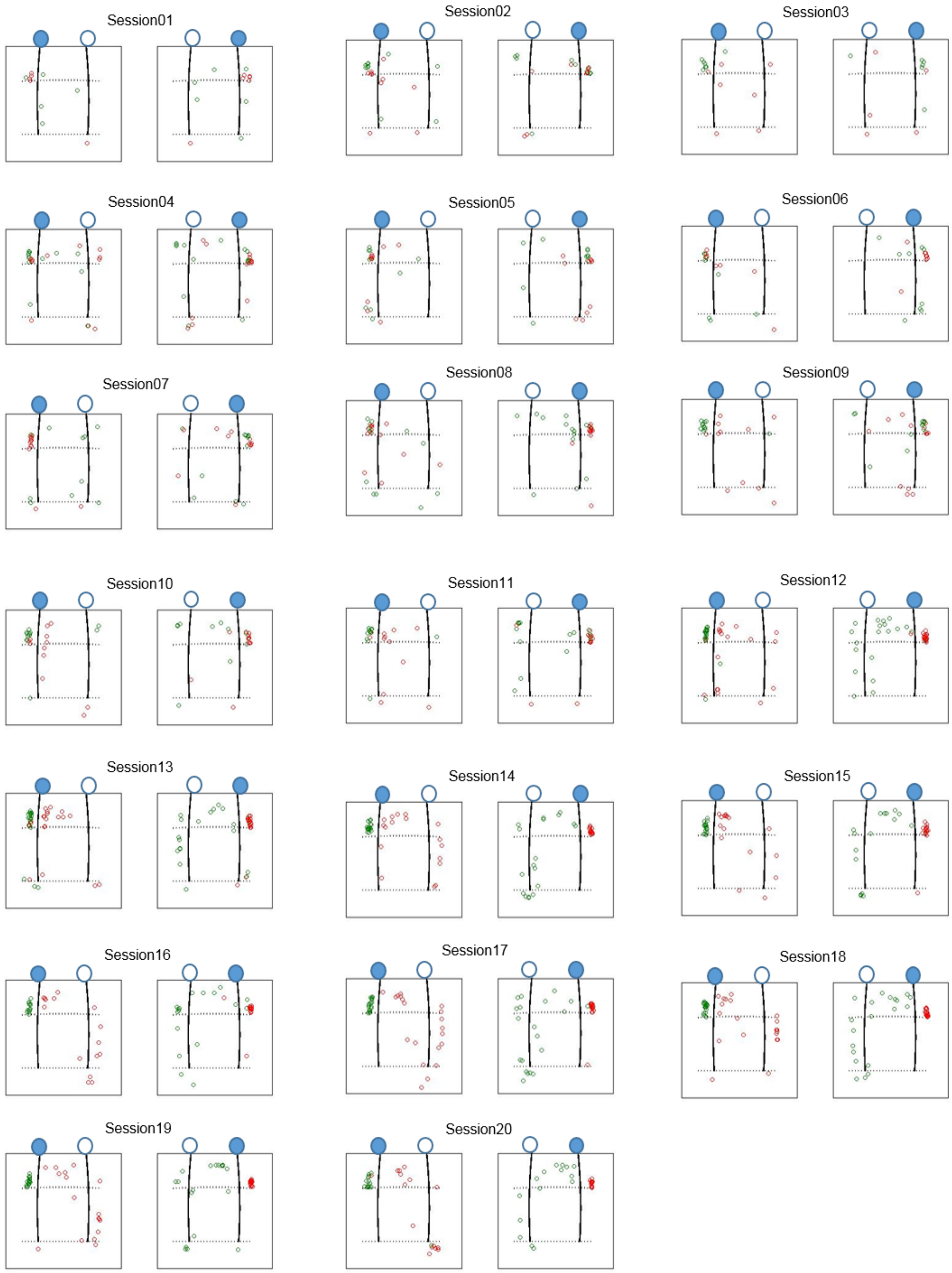

Pair #15

Figure S7

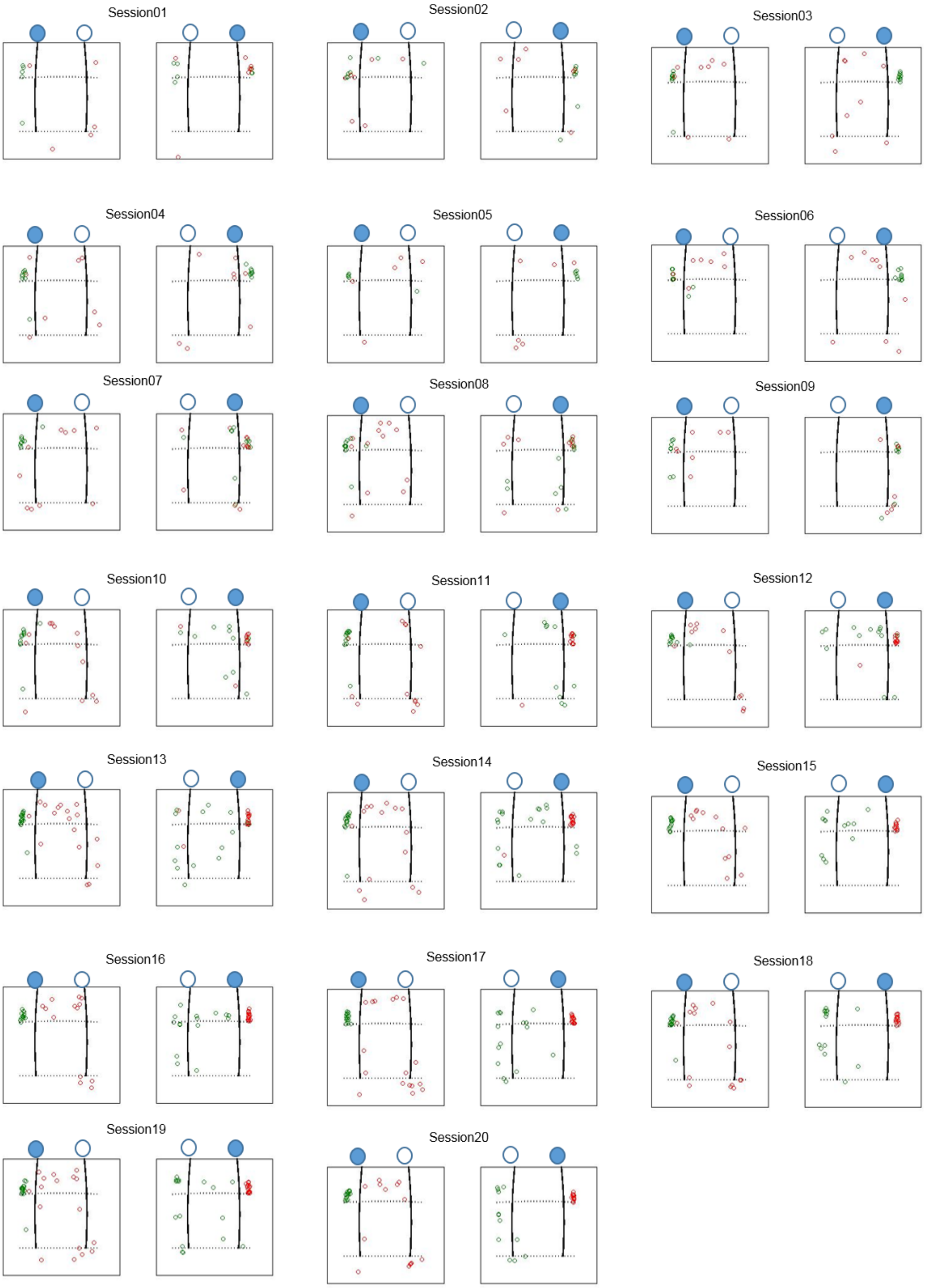

Pair #16

Figure S7

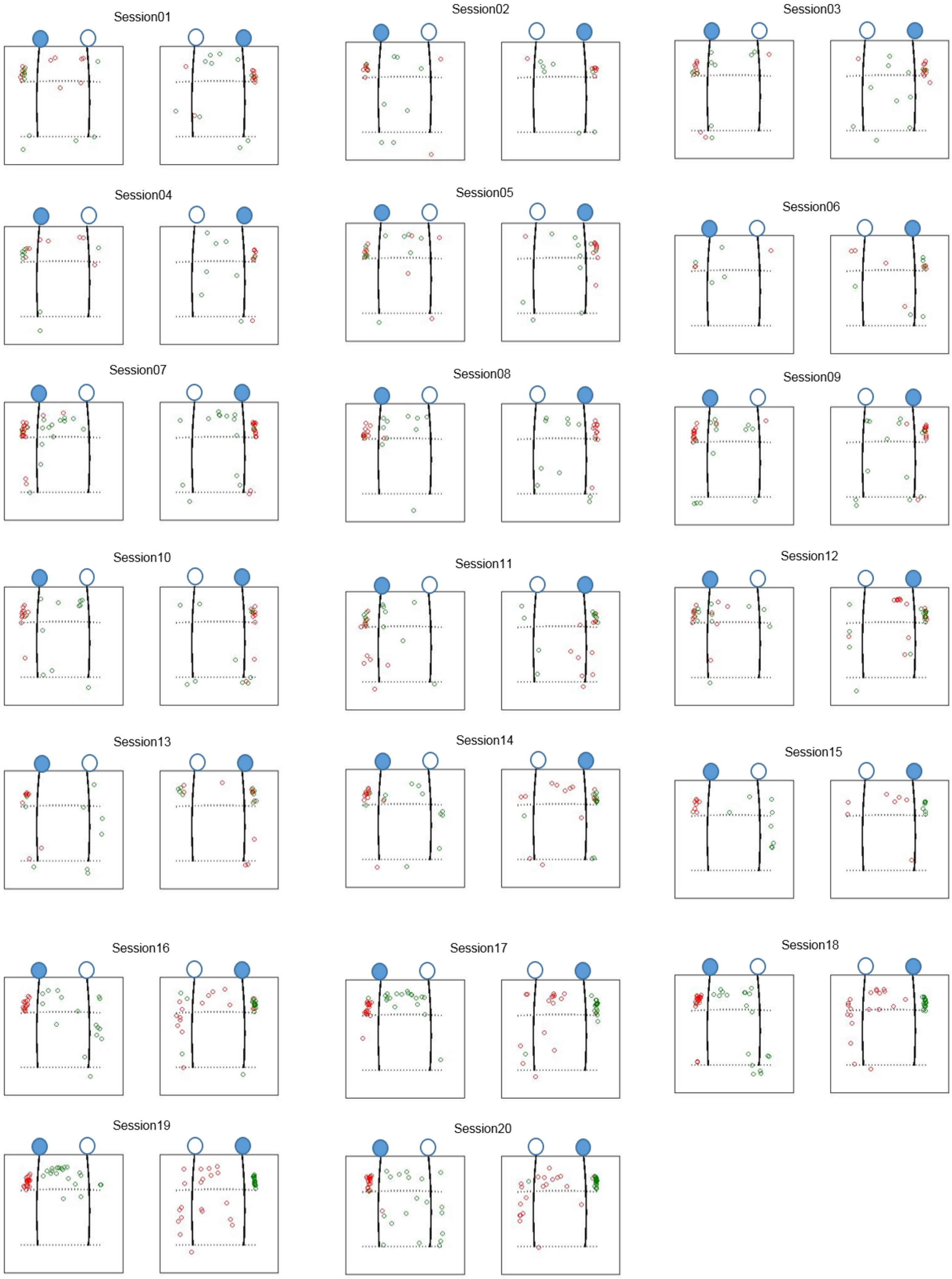

Pair #17

Figure S7

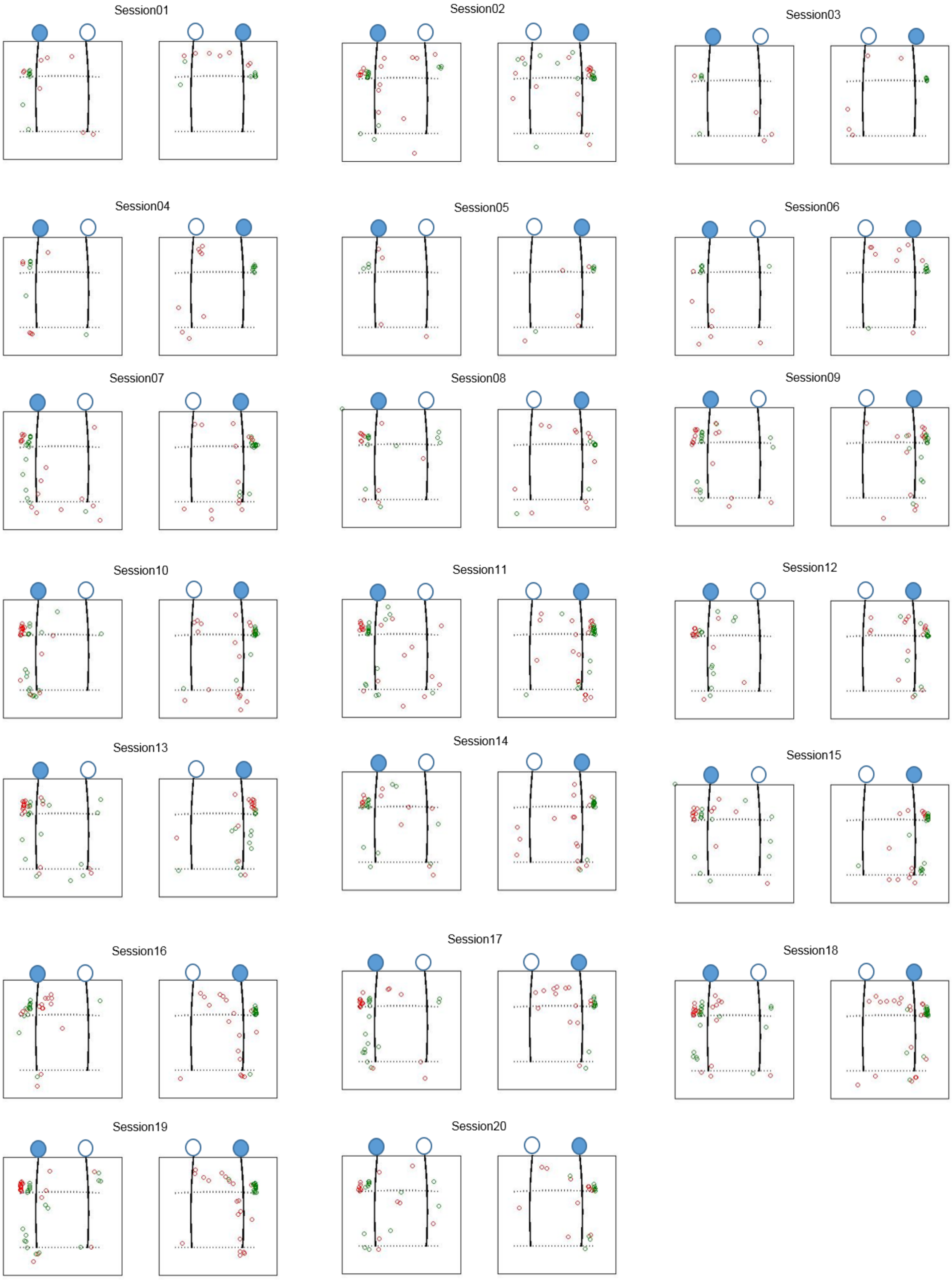

Pair #18

Figure S7

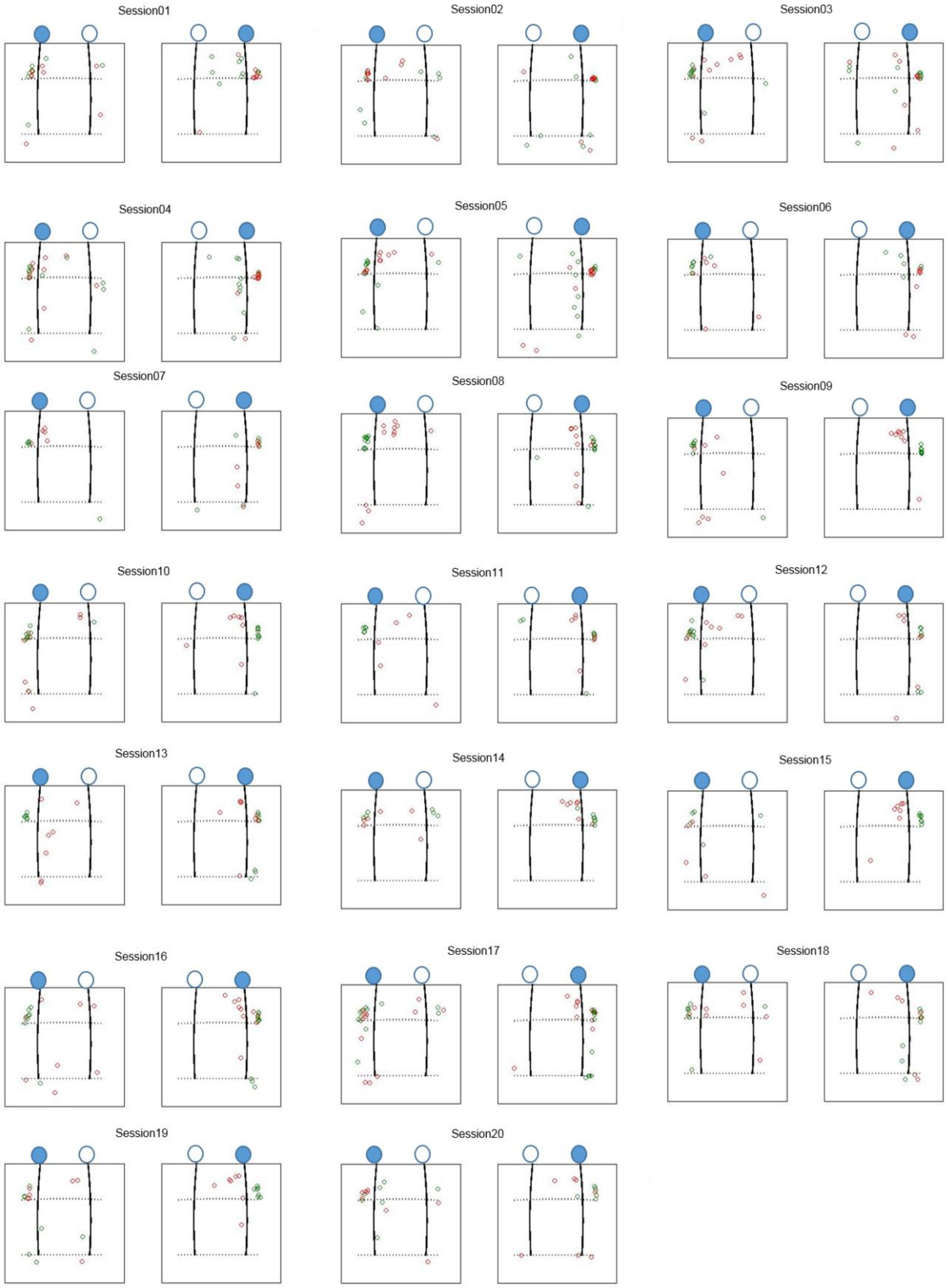

Pair #19

Figure S7

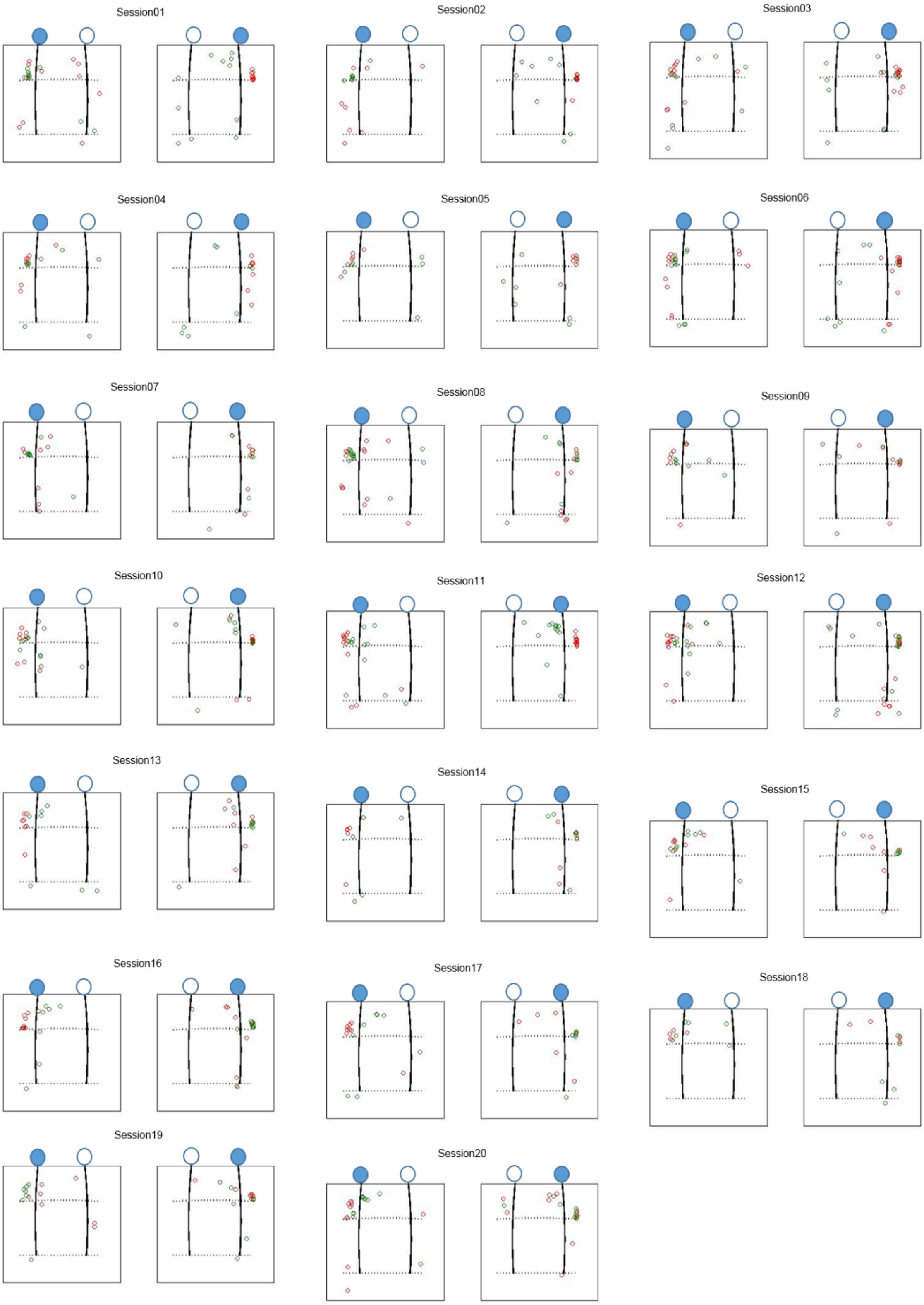

n=10	6sec		2sec		%visit6sec	
Day	mean	SE	mean	SE		
1	5.1	0.887568	6.4	0.921352	0.54	0.058564
2	7.9	1.779201	8.3	1.686877	0.64	0.073879
3	9.5	1.808928	8	1.498147	0.67	0.042881
4	9.9	1.386042	6.9	1.16857	0.64	0.073464
5	12.1	1.93477	7.3	1.813836	0.76	0.052988
6	14.3	2.011357	5.7	2.011357	0.85	0.045

n=10	6sec		4sec		%visit6sec	
Day	mean	SE	mean	SE		
1	7.4	0.968389	8.9	1.17804	0.45	0.051474
2	9.4	1.752459	9.4	1.661325	0.50	0.086968
3	12.9	1.797838	7.1	1.797838	0.65	0.089892
4	12.4	2.181742	7.4	2.082733	0.62	0.108432
5	13.7	1.751507	6.3	1.751507	0.69	0.087575
6	13.9	1.888268	6.1	1.888268	0.70	0.094413

Supplementary Table 1. Mice distinguish longer from shorter duration of WBS reward, visiting more to the longer duration arm, conforming to the matching rule. Top, 6-seconds vs. 2-seconds of WBS reward. Bottom, 6-seconds vs. 4-seconds of WBS reward.

Left cue		WBS-ON			WBS-OFF		
Pair type	Pair#	correct arm	center zone	incorrect arm	correct arm	center zone	incorrect arm
Mobs-Mobs	1	1.0	88.9	10.1	15.6	56.7	27.8
	2	2.0	88.8	9.2	8.7	60.9	30.4
	3	1.0	86.0	13.0	13.5	71.9	14.6
	4	2.0	92.0	6.0	24.6	40.6	34.8
	5	1.0	82.3	16.7	21.0	27.2	51.9
	6	5.1	92.9	2.0	22.5	48.8	28.8
	7	2.7	68.9	28.4	9.2	63.1	27.7
	8	1.0	88.0	11.0	16.1	24.2	59.7
Average		2.0	86.0	12.0	16.4	49.2	34.4
SEM		0.5	2.5	2.6	2.0	5.7	4.8
Mvio-Mvio	16	4.2	74.0	21.9	19.7	57.7	22.5
	17	75.6	11.6	12.8	75.8	18.2	6.1
	18	72.1	18.6	9.3	79.2	12.5	8.3
	19	73.7	18.4	7.9	86.8	5.3	7.9
Average		56.4	30.7	13.0	65.4	23.4	11.2
SEM		15.1	12.6	2.7	13.3	10.2	3.3

Supplementary Table 2. The position of the opponent mouse at the time point when the WBS reward was initiated or terminated. In M_{Obs}-M_{Obs} pairs, at the reward initiation time, the majority of the opponents remained in the center zone (86.0 ± 2.5%) and very few in the correct arm (2.0 ± 0.5%). At the reward termination time, five seconds later, a large proportion of mice who were staying in the center area moved out. The majority of them moved into the incorrect arm, in effect to stay away from the correct arm where the partner receives the reward. In the M_{Vio}-M_{Vio} pairs, even at the reward initiation time, the majority of the opponents were in the correct arm (56.4 ± 15.1%). At the reward termination time, even more mice were positioned in the correct arm (65.4 ± 13.3%).
