# Supplementary material for: Mice in social conflict show rule-observance behavior enhancing long-term benefit
Source: Nat Commun. 2017 Nov 7;8:1176. doi: 10.1038/s41467-017-01091-5 (PMC5673895; doi:10.1038/s41467-017-01091-5)
Supplement: Supplementary file 3 — Description of Additional Supplementary Files [file 41467_2017_1091_MOESM3_ESM.pdf]

File Name: Supplementary Movie 1
Description: Operant conditioning by food.

File Name: Supplementary Movie 2
Description: Operant conditioning by WBS. WBS was visualized as blinking light in the movie. However, actual WBS was invisible to the mouse.

File Name: Supplementary Movie 3
Description: Social interaction of mice conditioned by food.

File Name: Supplementary Movie 4
Description: Social interaction of mice conditioned by WBS.

File Name: Supplementary Movie 5
Description: A sample video of the conflict resolution test for food.

File Name: Supplementary Movie 6
Description: A sample video of the conflict resolution test for WBS.
